# Influence of periparturient and postpartum diets on rumen methanogen communities in three breeds of primiparous dairy cows

**DOI:** 10.1186/s12866-016-0694-7

**Published:** 2016-05-04

**Authors:** Laura M. Cersosimo, Melissa L. Bainbridge, Jana Kraft, André-Denis G. Wright

**Affiliations:** Department of Animal and Veterinary Sciences, The University of Vermont, 570 Main Street, Burlington, Vermont 05405 USA; Present Address: School of Animal and Comparative Biomedical Sciences, The University of Arizona, 1117 East Lowell Street, Tucson, AZ 85721 USA

**Keywords:** Archaea, Diversity, Holstein, Holstein-Jersey, Jersey, 16S rRNA gene, mcrA, Volatile fatty acids

## Abstract

**Background:**

Enteric methane from rumen methanogens is responsible for 25.9 % of total methane emissions in the United States. Rumen methanogens also contribute to decreased animal feed efficiency. For methane mitigation strategies to be successful, it is important to establish which factors influence the rumen methanogen community and rumen volatile fatty acids (VFA). In the present study, we used next-generation sequencing to determine if dairy breed and/or days in milk (DIM) (high-fiber periparturient versus high-starch postpartum diets) affect the rumen environment and methanogen community of primiparous Holstein, Jersey, and Holstein-Jersey crossbreeds.

**Results:**

When the 16S rRNA gene sequences were processed and assigned to operational taxonomic units (OTU), a core methanogen community was identified, consisting of *Methanobrevibacter* (*Mbr.) smithii, Mbr. thaueri, Mbr. ruminantium*, and *Mbr. millerae*. The 16S rRNA gene sequence reads clustered at 3 DIM, but not by breed. At 3 DIM, the mean % abundance of *Mbr. thaueri* was lower in Jerseys (26.9 %) and higher in Holsteins (30.7 %) and Holstein-Jersey crossbreeds (30.3 %) (*P* < 0.001). The molar concentrations of total VFA were higher at 3 DIM than at 93, 183, and 273 DIM, whereas the molar proportions of propionate were increased at 3 and 93 DIM, relative to 183 and 273 DIM. Rumen methanogen densities, distributions of the *Mbr.* species, and VFA molar proportions did not differ by breed.

**Conclusions:**

The data from the present study suggest that a core methanogen community is present among dairy breeds, through out a lactation. Furthermore, the methanogen communities were more influenced by DIM and the breed by DIM interactions than breed differences.

## Background

In the United States, enteric methane emissions from ruminants are the second largest anthropogenic source of methane, contributing to 25.9 % of all methane emissions and global warming [1]. Methane production caused by rumen archaea (i.e., methanogens) leads to a 2–12 % net loss of the dairy cow’s gross energy intake [2]. This loss contributes to a significant economic loss for farmers as it increases the quantity of feed needed to meet milk production demands.

The rumen is an anaerobic environment that houses a microbiome consisting of bacteria, protozoa, fungi, phages, and archaea. The bacteria, protozoa, and fungi (e.g., yeast) ferment feedstuff consumed by the host, and produce VFA. Acetate, butyrate, and propionate, the predominant VFA in the rumen, are the main energy sources for the host animal. Fermentation byproducts such as carbon dioxide, formate, hydrogen gas, methanol, and methylamines are used by methanogenic archaea for methane production. The majority of the methane is eructated and exhaled out by the ruminant into the environment. For methane mitigation strategies to be successful, it is important to identify factors that may influence the rumen environment and thus, affect the methanogen density and diversity.

In dairy cattle rumen digesta, methanogens belonging to the genus *Methanobrevibacter* (*Mbr.*) are the most abundant species and primarily use hydrogen and carbon dioxide as substrates for methanogenesis [3–5]. Methanogens from the genera *Methanosphaer*a (*Msp.*) and *Methanosarcina* use methanol and methylamines as substrates and are less abundant in the rumen [4]. Most *Mbr.* species in the rumen branch into two taxonomic clades, consisting of *Mbr. smithii, Mbr. gottschalkii, Mbr. millerae,* and *Mbr. thaueri* (i.e., *smithii*-*gottshcalkii*-*millerae*-*thaueri* (SGMT) clade), or *Mbr. ruminantium* and *Mbr. olleyae* (*ruminantium-olleyae* (RO) clade) [4]. Previous 16S rRNA gene sequence clone library data suggest that dairy breed influences the RO and SGMT clade distributions in the rumen [4].

Holstein and Jersey dairy cattle are the two most common dairy breeds used in the United States. Holstein cows are recognized for their high milk production, whereas Jersey cows are recognized for their increased fertility and higher milk components. Additionally, there is global interest in Holstein-Jersey crossbreeds to compensate for the decreased fertility in Holsteins and milk production in Jersey cows. It has been demonstrated that first generation Holstein-Jersey crosses have dry matter intakes, milk yields and solids in between those measured in Holstein and Jersey cows, respectively [6].

The transition period from a diet high in neutral detergent fiber (NDF) to a diet high in starch is a challenge for lactating dairy cattle. Prior to parturition, the NDF content in the diet is elevated, while after parturition the energy content is increased with higher starch and fat levels. Kumar et al*.* [7] showed no difference in archaeal Shannon diversity or taxa when cows were transitioned from a high-fiber pre-partum diet to a low-fiber post-partum diet, however, no studies described the rumen methanogen community across a lactation period. When quantifying VFA, another study observed that concentrations of total VFA, acetate, and propionate were decreased during the transition period in comparison to 100 days in milk (DIM) [8].

Previous research focused on rumen bacteria in pre- and post-partum dairy cattle, but rumen methanogens have not been identified or quantified under these conditions. Furthermore, the rumen methanogens of Holstein and Jersey dairy cattle with different parities and DIM were identified with limited data generated from pooled PCR samples using clone libraries. The present study focused on Holstein-Jersey crossbreeds, used next-generation sequencing (NGS), and animals of the same age, DIM, and parity. Given previous investigations into the rumen methanogen community in relation to breed and what is known about transitioning dairy cattle from one diet to another, we hypothesized that the rumen methanogen diversity and rumen VFA proportions in primiparous dairy cattle are affected by both breed and DIM, while methanogen densities do not vary. The objectives of the present study were to (1) measure the rumen VFA, (2) use NGS techniques to identify rumen methanogens, (3) distribute the archaeal 16S rRNA gene sequence reads into operational taxonomic units (OTU), (4) quantify the rumen methanogens, and (5) correlate VFA with specific rumen methanogen taxa from each breed during early (3 DIM), peak (93 DIM), mid- (183 DIM), and late-lactation (273 DIM).

## Results

The 16S rRNA gene sequence data set is accessible through NCBI’s Sequence Read Archive, under the study accession number [SRP058775].

### Rumen volatile fatty acids

Breed and breed x DIM differences in total VFA concentrations or in individual VFA molar proportions were not observed. Total VFA concentrations were highest at 3 DIM (*P* < 0.01). Propionate proportions were lowest at 273 DIM and highest at 3 and 93 DIM (*P* < 0.05). Relative to 3 DIM, acetate proportions were higher at 183 and 273 DIM (*P* <0.001). Isobutyrate and lactate proportions were highest at 273 DIM (*P* < 0.01). Isovalerate proportions did not differ by DIM (Table 1).

**Table 1:** Rumen volatile fatty acids from lactating Holstein, Jersey, Holstein-Jersey crossbreed dairy cows at 3, 93, 183, and 273 days in milk

VFA (% total)^a^	Days in milk				
	3	93	183	273	SE
Acetate	65.30 c	66.71 b	67.75 ab	68.53 a	0.51
Propionate	18.55 a	18.01 a	16.07 b	14.71 c	0.41
Butyrate	9.98 ab	9.52 b	10.22 a	10.01 ab	0.27
Isobutyrate	0.83 b	0.86 b	0.87 b	1.06 a	0.03
Valerate	0.93 ab	0.99 a	1.01 a	0.82 b	0.04
Isovalerate	0.84 a	0.72 a	0.70 a	0.76 a	0.05
Lactate	3.44 b	3.29 b	3.38 b	4.12 a	0.15
A:P^b^ ratio	3.59 c	3.80 c	4.26 b	4.69 a	0.12
Total VFA^c^ (mM)	144.5 a	112.8 b	118.7 b	105.2 b	6.41

^a^Means are based on Holstein (*n* = 7), Jersey (*n* = 8), and Holstein-Jersey crossbreed (*n* = 7)

Means within a row without a common letter differ (*P* < 0.05); ^b^ acetate:propionate; ^c^ volatile fatty acids

### Rumen methanogen densities

The rumen methanogen densities (log_10_ copy number of methyl-coenzyme M reductase A (mcrA) gene/mL whole rumen digesta) were not different by breed (*P* = 0.93) or DIM (*P* = 0.25). The mean and standard error (SE) of densities by breed were 6.51 ± 0.04 (Holsteins), 6.53 ± 0.04 (Jerseys), and 6.52 ± 0.04 for (Holstein x Jersey crossbreeds), while the densities by DIM were 6.48 ± 0.05 (3 DIM), 6.49 ± 0.05 (93 DIM), 6.61 ± 0.05 (183 DIM), and 6.49 ± 0.05 (273 DIM), respectively. 

### Bioinformatics analyses of the rumen methanogen community

**Fig. 1:**
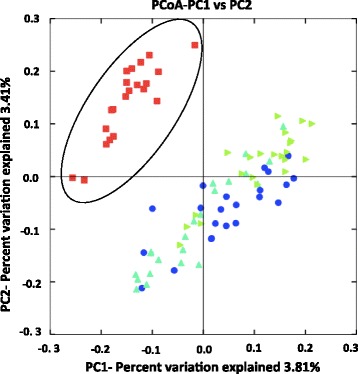
Principal coordinate analysis (PCoA) of rumen methanogen 16S rRNA sequences across a lactation. The PCoA demonstrates the clustering of 16S rRNA sequences from Holstein (*n* = 7), Jersey (*n* = 8), and Holstein-Jersey crossbreed (*n* = 7) cows at 3, 93, 183, and 273 days in milk (DIM). Red squares represent 3 DIM, the blue circles represent 93 DIM, mint green triangles represent 183 DIM, and light green triangles represent 273 DIM

After 8,248,879 raw 16S rRNA sequence reads were quality checked, a total of 1,822,214 sequences from 87 whole rumen digesta samples had a Phred score of 25 or greater. The final data set contained 1,683,569 non-chimeric 16S rRNA gene sequence reads. 16S rRNA gene sequence read lengths (not including dashes) ranged from 357–390 bp with a mean length of 358 bp. The mean and SE of sequence reads per individual by breed were: 19,039 ± 2,667 (Holsteins), 19,096 ± 2,744 (Jerseys), and 19,947 ± 3,034 (Holstein x Jersey crossbreeds). There were 298,689 total unique sequences (17.7 % of total reads) with 67,072 chimeras removed. The numbers of unique sequence reads for each time point was: 67,111 (3 DIM), 87,455 (93 DIM), 77,477 (183 DIM), and 66,646 (273 DIM). Principle coordinate analysis (PCoA) did not demonstrate clustering of methanogen communities by breed. However, rumen methanogen communities clustered at 3 DIM, but not at other time points (Fig. 1).

All sequence reads belonged to the phylum Euryarchaeota. The SGMT and RO clades did not differ by breed or DIM (Table 2). The majority of the total sequences reads were related to four methanogen species, *Mbr. smithii*, *Mbr. thaueri*, *Mbr. ruminantium*, and *Mbr. millerae* (Table 2). Because there was a significant interaction between breed and DIM for *Mbr. thaueri, Mbr. millerae, Methanosphaer*a*,* and Methanoplasmatales, breed differences at specific DIM are presented. At 3 DIM, the mean % abundance of *Mbr. thaueri* was lower in Jerseys (26.9 %) and higher in Holsteins (30.7 %) and Holstein-Jersey crossbreeds (30.3 %) (*P* < 0.001). At 93 DIM, a lower abundance of the species *Mbr. thaueri* was observed in Holsteins (24.5 %, *P* < 0.05) and Holstein-Jersey crossbreeds (19.4 %, *P* < 0.01) when compared to Jerseys (35.0 %). The species, *Mbr. ruminantium* (*P* < 0.05) was higher at 93 DIM than at 273 DIM. *.* The less abundant methanogen species (<5 %) *Mbr. gottschalkii* and *Mbr. woesei* also varied by DIM, but not by breed*.* At 93 DIM (*P* < 0.05) and 273 DIM (*P* < 0.001), the abundance of *Mbr. gottschalkii* was higher than at 3 DIM. *Mbr. woesei* (*P* <0.001) were more abundant at 3 DIM, whereas the order Methanosarcinales was more abundant at 183 DIM (*P* < 0.01). Less than 1 % of total methanogen sequences were distantly related to the following methanogen genera: *Methanoculleus, Methanolobus Methanoplanus, Methanospirillium,* and *Methanosarcina.*

**Table 2:** Classification of rumen methanogen 16S rRNA sequence reads to taxa from Holstein, Jersey, Holstein-Jersey crossbreed dairy cows at 3, 93, 183, and 273 days in milk

DIM^b^	3			93			183			273			SE	Significance^a^		
Breed	H	J	X	H	J	X	H	J	X	H	J	X		B	DIM	B x DIM
*Methanobrevibacter*	99.30	96.15	99.42	98.08	99.02	99.10	97.28	98.25	94.81	99.23	98.39	98.87	0.99	NS	†	NS
*Mbr* ^*c*^ *woesei*	2.12	2.47	1.58	1.03	1.10	1.12	0.59	0.86	0.69	1.06	0.94	1.03	0.22	NS	***	NS
*Mbr. smithii*	28.55	28.09	35.72	27.12	29.75	36.21	32.72	30.72	31.90	27.25	32.57	35.91	3.64	†	NS	NS
*Mbr. gottschalkii*	0.04	0.02	0.05	0.80	0.11	0.28	0.25	0.14	0.21	0.80	0.19	0.30	0.17	NS	*	NS
*Mbr. millerae*	10.99	8.39	8.0	5.17	7.04	7.80	7.45	8.55	6.08	5.02	8.72	7.45	1.15	NS	*	*
*Mbr. thaueri*	30.70	26.70	30.32	24.51	35.03	19.43	28.03	32.34	26.93	35.30	37.09	36.36	3.51	NS	*	***
SGMT Clade	70.29	63.15	74.49	57.45	71.93	63.71	68.45	71.74	65.13	68.37	78.56	80.02	5.81	NS	NS	NS
*Mbr. ruminantium*	26.18	29.82	22.88	38.73	25.31	33.45	27.43	24.92	28.38	29.01	18.41	17.24	5.41	NS	†	NS
*Mbr. olleyae*	0.36	0.37	0.19	0.48	0.35	0.46	0.47	0.35	0.33	0.44	0.23	0.29	0.08	NS	NS	NS
RO Clade	26.54	30.02	23.07	39.21	25.66	33.92	27.91	25.29	28.71	29.45	18.64	17.53	5.50	NS	†	NS
*Methanosphaera*	0.41	0.43	0.42	1.01	0.41	0.74	0.50	0.38	0.64	0.46	0.41	0.28	0.10	†	**	*
Methanoplasmatales	0.15	2.02	0.07	0.49	0.15	0.03	0.90	0.31	1.93	0.22	0.41	0.45	0.45	NS	NS	*
Methanosarcinales	0.08	1.33	0.08	0.35	0.37	0.12	1.16	0.99	2.27	0.01	0.55	0.19	0.51	NS	**	NS

^a^ *P*-value of the effects due to breed (Holstein, Jersey, and Holstein-Jersey crossbreed) and their interaction and comparison to time period (3, 93, 183, and 273 DIM)

^b^Days in milk (DIM), H = Holstein (*n* = 7), J = Jersey (*n* = 8), X = Holstein-Jersey crossbreed (*n* = 7), Breed (*B*), ^c^ *Mbr* = *Methanobrevibacter*

*** *P* < 0.001; ** *P* < 0.01; * *P* < 0.05; † 0.05 ≤ *P* ≤ 0.10; no significance (NS) *P* > 0.10

### OTU-based analyses

The 16S rRNA gene sequence reads clustered into 403 (3 DIM), 383 (93 DIM), 590 (183 DIM), and 547 OTUs (273 DIM). Breed and breed by DIM did not affect rumen methanogen diversity measures (Table 3). Good’s coverage, Shannon diversity and Inverse Simpson indices were affected by DIM. The Inverse Simpson indices were highest at 3 and 183 DIM (*P* < 0.01), while Good’s coverage and the Shannon Diversity indices were highest at 3 DIM (*P* < 0.05). The most and least OTUs shared between all animals were at 93 and 273 DIM, respectively (Table 4).

**Table 3:** Operational taxonomic unit-based diversity measurements from Holstein, Jersey, Holstein-Jersey crossbreed dairy cows at 3, 93, 183, and 273 days in milk

DIM	3			93			183			273			SE	Significance^a^	
Breed^b^	H	J	X	H	J	X	H	J	X	H	J	X		B	DIM
OTU	23.7	37.7	18.5	34.3	33.5	29.7	28.9	22.2	31.1	20.0	24.4	23.0	1.4	NS	NS
Coverage (%)	99.8	99.7	99.9	99.8	99.8	99.8	99.4	99.6	99.4	99.7	99.5	99.6	<0.1	NS	***
Shannon Diversity	1.5	1.5	1.4	1.4	1.4	1.3	1.4	1.4	1.5	1.3	1.4	1.4	<0.1	NS	*
Inverse Simpson	3.8	3.6	3.4	3.1	3.3	3.0	3.2	3.5	3.7	3.1	3.3	3.2	0.1	NS	**
Chao I estimator	52.0	94.7	32.1	68.4	63.4	59.2	82.0	47.0	61.7	54.1	52.2	54.6	4.2	NS	NS

^a^ *P*-value of the effects due to breed (Holstein, Jersey, and Holstein-Jersey crossbreed) and their interaction and comparison to time period (3, 93, 183, and 273 DIM)

^b^ H = Holstein (*n* = 7); J = Jersey (*n* = 8), X = Holstein-Jersey crossbreed (*n* = 7), Breed (*B*), Days in milk (*DIM*), *** *P* < 0.001; ** *P* < 0.01;* *P* < 0.05; no significance (NS) *P* > 0.10

**Table 4:** Number of shared operational taxonomic units among and between Holstein, Jersey, Holstein-Jersey crossbreed dairy cows at 3, 93, 183, and 273 days in milk

	Number of shared OTUs^a^			
	3 DIM^b^	93 DIM	183 DIM	273 DIM
Holstein	13	16	11	10
Jersey	12	14	12	9
Holstein x Jersey	11	13	11	9
Between H-J^c^	12	13	11	9
Between H-X	11	13	12	8
Between J-X	10	14	12	8
All	10	13	11	8

^a^Operational taxonomic unit; ^b^Days in milk; ^c^ H = Holstein (*n* = 7); J = Jersey (*n* = 8), X = Holstein-Jersey crossbreed (*n* = 7),

The top four OTUs shared by all breeds and at each stage of lactation were related to the species *Mbr. smithii, Mbr. thaueri*, *Mbr. ruminantium*, and *Mbr. millerae*. The least abundant OTUs were related to *Mbr. wolinii, Mbr. gottschalkii, Mbr. olleyae, Mbr. arboriphilus, Msp. stadtmanae,* unclassified *Methanosarcina,* Methanoplasmatales, *Methanoculleus*, and *Methanolobus*. The majority of the sequence reads (98.7 ± 0.1 %) clustered into OTU 1–4. At 3, 93, and 183 DIM, the mean abundance of OTU 1 was 30.8 ± 2.0 %, 30.9 ± 2.8 %, and 31.7 ± 1.6 %, respectively. At 273 DIM, OTU 2 was most abundant with 36.1 ± 2.3 %. No breed effects were observed for OTUs 1–4 and the least abundant OTUs. However, the abundance of OTU 1 was lowered (*P* < 0.05) in Holsteins (28.8 ± 2.0 %) when compared to Holstein x Jersey crossbreeds (34.9 ± 2.0 %). DIM did not affect the distribution of OTU 3 or the least abundant OTUs. The abundance of OTU 2 increased at 273 DIM (*P* < 0.05), while the abundance of OTU 4 increased at 3 DIM (*P* <0.05).

### Relationship between methanogen taxa and VFA

**Fig. 2:**
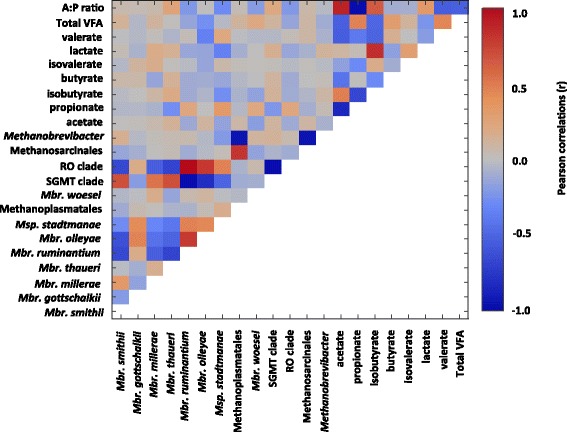
Pearson correlation heatmap comparing the abundance of rumen methanogen taxa to rumen VFA. The heatmap depicts correlations made between rumen methanogen taxa and VFA from primiparous Holstein (*n* = 7), Jersey (*n* = 8), and Holstein-Jersey crossbreed (*n* = 7). acetate to propionate (A:P), Methanobrevibacter (*Mbr.*), *Methanosphaera* (*Msp.*) *smithii*-*gottshchalkii*-*millerae*-*thaueri* (SGMT), *ruminantium-olleyae* (RO), volatile fatty acids (VFA)

Notably, the SGMT and RO clades were negatively correlated (*r* = −0.98, *P* < 0.0001) (Fig. 2). A negative correlation between *Mbr. smithii* and *Mbr. ruminantium* was observed (*r* = −0.66, *P* < 0.0001). The abundance of the order Methanosarcinales was positively correlated with the order Methanoplasmatales (*r =*0.81, *P* < 0.0001). Several weak correlations were observed between most methanogen taxa and VFA (Fig. 2). The species, *Msp. stadtmanae* was negatively correlated to lactate (*r* = −0.34, *P* < 0.01) and positively correlated to propionate (*r* = 0.33, *P* < 0.01). Propionate was positively and negatively correlated to *Mbr. ruminantium* (*r* = 0.22, *P* = 0.04) and *Mbr. thaueri* (*r* = −0.27, *P* = 0.04), respectively. Several correlations were observed between individual VFA. Acetate was negatively correlated to propionate (*r* = −0.84, *P* < 0.001), butyrate (*r* = −0.42, *P* < 0.001), and valerate (*r* = −0.54, *P* < 0.001) and positively correlated to isobutyrate (*r* = 0.48, *P* < 0.001). Propionate was negatively correlated to isobutyrate (*r* = −0.66, *P* < 0.001) and lactate (*r* = −0.39, *P* < 0.001) and positively correlated to valerate (*r* = 0.50, *P* < 0.001).

## Discussion

The present study is the first to investigate the rumen methanogen community across a lactation period in three dairy cattle breeds. The purpose of this experiment was to provide more knowledge about the rumen methanogen community and rumen parameters at 3, 93, 183, and 273 DIM in Holstein, Jersey, and Holstein-Jersey crossbreeds. This study identified the core methanogen community with NGS technologies, quantified rumen VFA and methanogen densities, and correlated rumen methanogen species to one another and to VFA.

### Volatile fatty acids

VFA are the main energy source provided to lactating dairy cattle and are the by products of carbohydrate fermentation by rumen bacteria, protozoa, and fungi. Generally, propionate is a precursor to glucose and is increased when animals are provided a high-starch diet or provided the ionophore, monensin. Relative to 183 and 273 DIM, proportions of propionate were increased at 3 and 93 DIM, suggesting a greater demand for glucose by the cow during early lactation. Although the animals were provided 0.06 % monensin pre-partum versus 0.02 % post-partum, it is not possible to correlate the increase in propionate at 3 and 93 DIM with this additive. An effect from monensin would be more plausible at 3 DIM, when the cows were transitioning from a pre-partum to a post-partum diet, but this would not explain why propionate was also increased at 93 DIM.

Furthermore, the increase in total VFA concentrations observed at 3 DIM suggests an increase in carbohydrate fermentation at the start of lactation. Johnson et al*.* [9] stated that the fermentation of fiber is favored, providing insight into why VFA concentrations were elevated at 3 DIM versus 93, 183, and 273 DIM. In contrast, Danielsson et al. [10] found that total VFA concentrations did not vary in cannulated mid-lactation dairy cattle consuming 500:500 and 900:100 g/kg dry matter forage to concentration diets.

The present study is the first to compare the methanogen densities in three breeds of dairy cattle and by DIM. In agreement with our hypothesis, the methanogen densities did not vary by breed or DIM. Previously reported methanogen densities (i.e., log_10_ mcrA gene copies) from bulls on high-fiber (9.02) and starch diets (9.07) [11] were higher than what was observed in our study [11]. However, differences between the methanogen densities were not observed between the two diet groups [11]. In a study by Zhou et al. [12], the use of an exogenous fibrolytic feed enzyme additive did not affect the methanogen densities, yet, affected the methanogen community and methane production of lactating Holstein cows. Therefore, it appears that methanogen densities are not markedly affected by these specific diet alterations.

Previous work in dairy [4, 7, 10] and beef cattle [11, 13] also showed the genus *Mbr* to be the most predominant genus. As methanogens belonging to the genus *Mbr* use the rumen fermentation byproducts, such as hydrogen and carbon dioxide as substrates for methanogenesis, it is thought that the high levels of these byproducts in the rumen enable these methanogens to thrive over other species that rely on scarce substrates such as methylamines, methanol, or acetate [14, 15].

Although *Mbr* is the most abundant archaeal genus in ruminants, there are several species that are distributed into two different phylogenetic clades (i.e., SGMT and RO). In the present study, the SGMT clade was the most dominant branch by breed and DIM. Both *Mbr. smithii* and *Mbr. thaueri* made up the majority of the SGMT clade, while *Mbr. ruminantium* made up the majority of the RO clade. Previous research, using the same archaeal forward primer (Met86F), suggested a difference between SGMT-RO clade distributions between Holstein and Jersey cows [4]. However, the study revealed several limitations in the interpretation of the results. Animals were not blocked by parity, DIM, or age, the PCR products were pooled by breed and a clone library was constructed for each breed, and a limited number of clones were sequenced. It is conceivable that these variables, but not primer bias, may have contributed to the observed breed differences. Another study showed a prevalence of the RO clade in both corn-fed Hereford crossbreed and potato-fed Hereford feedlot cattle in Canada [13]. Finally, the present study showed a strong negative correlation between the two clades suggesting that ruminants possess either a high abundance of SGMT or of RO and that dairy breed and DIM do not impact these proportions.

Because the four methanogen species *Mbr. smithii*, *Mbr. thaueri*, *Mbr. ruminantium*, and *Mbr. millerae* were identified in each breed and at each DIM time point investigated, our data showed the presence of a core methanogen community. Jeyanathan et al. [16] identified a common methanogen community between Holstein-Jersey crossbreeds, sheep, and red deer. Finding a core rumen methanogen community will enable further investigations into targeting specific species that are key contributors to methane production. Future work could isolate these species and determine which species produce the most methane.

Three out of the four methanogen species in the present study were previously identified in both Holstein and Jersey cows, while *Mbr. thaueri* was not. Recently, *Mbr. thaueri* was identified with the same primer pair used in the present study, at a high abundance in wild impalas from South Africa [17]. Omission of *Mbr. thaueri* from previous studies could be due to lack of sequencing depth or diet of the animal. The higher abundance of *Mbr. thaueri* in Jersey cows at 93 DIM (i.e., peak lactation) may be a result of a higher dry matter intake (DMI), but future studies are needed to draw a clear link between DMI, milk yield, and the rumen methanogen species identified. It is conceivable that *Mbr. thaueri* and the other three methanogen species persisted because the rumen environment and the substrates created by bacteria, protozoa, and fungi enabled these methanogens to thrive.

Throughout the lactation period and by breed, the methanol-utilizing genus, *Msp.* was identified in low abundances. Its mean % abundance was highest around peak lactation (93 DIM) and lowest at 3, 183, and 273 DIM. Similarly, Kumar et al. [7] compared the methanogen diversity between Holsteins at four weeks before calving and 1–5 days after calving and observed no differences in the genera *Mbr* or *Msp.* At 1–5 DIM, the methanol-utilizing genus *Msp* was more abundant (4.5 %) in primiparous Holsteins than those from the present study (<1 %). Like in the present study, animals were stomach tubed 2–3 h post-feeding and received a diet before calving with the same NDF content (44 %, [7]). *Msp* is typically more abundant in animals consuming feeds with elevated pectin levels [14]. Although not analyzed, it is possible that the diet in the present study had a lower quantity of pectin.

Although both OTU and 16S rRNA gene sequence classifications identified a core methanogen community, certain methanogen species were more abundant at different DIM time points. Relative to 93, 183, and 273 DIM the proportions of *Mbr. millerae* and *Mbr. woesei* were highest at 3 DIM. While previous research has not focused on rumen methanogen communities pre- and post-partum, one study suggested that the highly cellulolytic anaerobic fungi were more prevalent in pre-partum dairy cows, while rumen protozoa were less prevalent [18]. This suggests that as the dairy cows transition to a diet typically fed post-partum, there is a shift in the rumen microbiome and likely in the methanogen community.

The OTU coverage in the present study was almost 100 %, indicating a sufficient sampling effort. OTU distribution did not vary by breed or DIM and the majority of the sequences were distributed into four main OTUs. The methanogen diversity in the present study was not influenced by breed, but by DIM. King et al. [4] reported a higher Shannon diversity index and number of OTUs in lactating Holstein cows than in Jersey cows. However, the limited number of sequences from the cloned libraries, pooled samples by breed, and parity may have influenced the results. According to Kumar et al. [7], multiparous Holstein cows exhibit a higher Shannon diversity index than primiparous.

The Shannon diversity indices (i.e., species evenness and abundance) from the present study were highest at 3 DIM, while the 16S rRNA gene sequences reads clustered during this time as well. At 93, 183, and 273 DIM the sequence reads were mixed in one cluster. Previous research also demonstrated that the Shannon diversity indices of methanogens increased in bulls on a high-fiber diet (0.95) and decreased with a high-starch diet (0.79) [11]. These data suggested that a high-fiber diet leads to a more diverse methanogen community when compared to a high-starch diet. In another study, the Shannon diversity of Holsteins at 4 weeks before calving and 1–5 days after calving did not differ, but was most likely because there was not enough time between sampling [7]. Belanche et al. [19] suggested that the increased amount of cellulose and other heteropolysaccharides in a diet high in fiber leads to a more diverse microbial community. Therefore, a more diverse bacterial, fungal, or protozoal community may provide different substrates that enable the presence of a more diverse methanogen community.

## Conclusions

The data presented here are the first to characterize the rumen methanogen communities in three dairy cattle breeds across a lactation period. NGS produced over 1 million sequence reads and demonstrated that diversity was different at 3 DIM. Notably, a core methanogen community persisted and consisted of four species, *Mbr. smithii, Mbr. thaueri, Mbr. ruminantium,* and *Mbr. millerae.* These methanogens may play a significant role in methanogenesis and in the utilization of substrates from bacterial, protozoal, and fungal fermentation. However future work is required to better delineate these relationships. The SGMT*-*RO clades did not vary by breed or DIM, instead, the SGMT clade was dominant in all three breeds. Although our results show that breed does not affect the rumen methanogen taxa *per se*, more studies are needed to clarify if this finding is consistent in other geographic locations and in dairy cattle consuming varying diets.

## Methods

### Animal sampling

From May 2013 to May 2014, 22 primiparous lactating dairy cattle (7 Holstein (H), 8 Jersey (J), and 7 first generation Holstein x Jersey crossbreeds (X)) were co-housed at the University of Vermont (UVM) Paul Miller Research Complex in South Burlington, VT. At 3 DIM, one Jersey was excluded from all data analyses because of post-partum health concerns. The Institutional Animal Care and Use Committee at the UVM approved all animal sample collection methods under protocol # 13–031. All animals calved within a 2-month period. At 3, 93, 183, and 273 DIM, whole rumen digesta samples (50 mL) were collected 2-3 h post-feeding at 0900 h via stomach intubation from each animal. To collect rumen samples, a flexible milk hose (2.54 cm diameter) was passed through a speculum to the esophagus and to the rumen. The hose was marked at 200 cm to indicate the approximate location of the rumen. Once the tube contacted the fiber mat and rumen sounds were heard, a 600 cc livestock drench gun (Labelvage, France) collected the digesta. Whole digesta samples were immediately frozen at −20 °C to minimize microbial activity.

### Diet

Prior to calving, all animals consumed a pre-partum total mixed ration (TMR) diet. Within 24 h post-partum, each cow was transitioned to a diet that was higher in starch and lower in NDF in comparison to the diet fed pre-partum (Table 5). Through out the study, a 70:30 forage to concentrate TMR was fed. Prior to calving the forages included corn silage (51.2 %), haylage (8.3 %), hay (13.4 %), and concentrate (27.2 %). The concentrate provided pre-partum contained: Amino Max® (Afgritech, Watertown, NY;18.8 %)(a mixture of 2.5 % dry matter (DM) lysine, 0.9 % methionine, and 16.3 % other essential amino acids derived from canola and soybean meals) soybean hulls (16.1 %), PastureChlor® (West Central, Ralston, IA;16.9 %) (0.5 % DM Ca, 6.1 % Mg, and 10.6 % Cl), canola meal (15.7 %), soybean meal (12.5 %), SoyChlor® (West Central, Ralston, IA;12.5 %) (high rumen bypass soybean meal with 4.5 % DM Ca, 2.8 % Mg, and 10.3 % Cl), calcium carbonate (4.4 %), magnesium sulfate (1.3 %), trace vitamins and minerals (0.7 %), magnesium oxide (0.5 %), sodium chloride (0.5 %), and Rumensin® (Elanco Animal Health, Greenfield, IN; 0.06 %). Both PastureChlor® and SoyChlor® are cation/anion supplements typically added to a pre-freshening dairy cow diet and are provided to prevent hypocalcemia (milk fever). After the animals calved, the forage consisted of corn silage (52.3 %), haylage (15.9 %), and concentrate (31.8 %). The concentrate provided post-partum contained: corn grain (24.6 %), citrus pulp (19.1 %), Amino Max® (16.4 %), soybean meal (16.4 %), canola meal (10.9 %), Amino Enhancer (Poulin Grain Inc., Newport, VT; 5.5 %) (blood and feather meal-derived amino acids with 8.0 % DM lysine and 1.1 % methionine), calcium carbonate (2.5 %), sodium sesquinate (2.2 %), sodium chloride (1.2 %), magnesium oxide (0.7 %), trace mineral premix and vitamins (0.43 %), zinc methionine (0.05 %), and Rumensin® (0.02 %). The ionophore, monensin (i.e., Rumensin) was provided in both diets to simulate the diet of a typical lactating dairy cow. Hook et al. [20] demonstrated that monensin does not alter the rumen methanogen diversity or density in lactating dairy cows. TMR samples were collected weekly for three consecutive days and composited at the end of each week. Because cows calved within two months of one another, the mean and the standard error of the diet provided before calving are reported. Cumberland Valley Analytical Services (Hagerstown, MD) analyzed the feed samples and provided nutrient composition data.

**Table 5:** Chemical composition of the diets (% DM basis) provided pre- and postpartum

Nutrient		Days in Milk			
	Pre-fresh	3	93	183	273
DM^a^ %	36.8 ± 1.2	38.9	40.7	41.3	43.0
CP^b^	14.1 ± 0.3	15.2	13.8	14.9	17.0
aNDFom^c^	35.0 ± 2.1	30.5	27.8	27.7	25.0
lignin	4.8 ± 0.2	4.3	5.0	4.0	4.7
starch	13.9 ± 4.0	21.3	22.4	25.3	21.7
sugar	3.1 ± 1.1	5.3	4.6	4.0	4.3

^a^DM- dry matter; ^b^CP- crude protein; ^c^aNDFom- ash-corrected neutral detergent fiber

### Volatile fatty acid analysis

The whole rumen digesta samples were spun in a Beckman J2-21 centrifuge at 10,000 x *g* for 20 min at 4 °C and the resulting supernatant was filtered through a 25 mm hardened ashless filter (Whatman Inc. Clifton, NJ) to remove debris. Subsequently, the samples were diluted 1:1 with 0.06 M oxalic acid containing 50 μM trimethyl acetic acid (internal standard) and analyzed for VFA by gas chromatography (Varian 3800 GC, Walnut Creek, CA) coupled with a flame ionization detector and a customized packed column (2 m x 2 mm ID glass) with 4 % carbowax and 80/120 Carbopac B-DA (Sulpeco, Bellefonte, PA) with nitrogen as carrier gas (15 mL/min flow rate). The other gases were purified air at 300 mL/min and hydrogen makeup gas at 30 mL/min. The column was operated at 175 °C; the total run time was 25 min. Both the injector and detector temperature were kept at 200 °C. The injection volume was 1 μL. The identification of VFA was based on retention times against known standards using software Star Chromatography v5 (Varian) and quantified for their concentrations using respective VFA standards. Results are expressed in mM VFA.

### Microbial DNA extraction

Across 4 time points (3, 93, 183, and 273 DIM), 87 individual whole rumen digesta samples were collected. The previously frozen samples were thawed overnight at 4 °C. Each sample was vortexed for 30s to homogenize the sample and break up the solid particles that settled to the bottom of the conical tube. The microbial DNA was extracted using the repeated-bead beating plus column (RBB + C) method [21] and followed previously described procedures [17].

### Real-time PCR amplification

The log_10_ of the copy number of the mcrA gene per mL of rumen digesta was determined by real-time PCR, each sample was amplified in triplicate. Each real-time PCR included: 12.5 μL of SYBR® Green Mix, 6.5 μL of double distilled water, 2.5 μL of the methanogen-specific primer pair, mcrA-F and mcrA-R [22], and 1 μL of either diluted template DNA (10 ng/μL), positive (mixture of microbial DNA extract from King et al. [4]) or negative controls (double-distilled water). The mcrA gene was amplified in a Bio-Rad C1000 Touch Thermal Cycler (Bio-Rad, Hercules, CA) under previously published conditions [22]. The five mcrA gene standards were created by serial dilutions of 10 ng/μL of purified PCR product. The range of concentrations for the standard curve was from 0.001–10 ng/μL. An acceptable standard curve had an R^2^ value greater or equal to 0.997. Using the BioRad CFX Manager (v.3.0) a model equation of the standard curve (*y* = mx + b) was used, where “x” was the log of the starting quantity, “b” the y-intercept of the quantification value (Cq), and “m” the slope of the line for log starting quantity versus Cq values. Individual densities were calculated using previously established methods [23].

### PCR Amplification of the 16S rRNA gene

The archaeal-specific primer pair, Met86F [24] and Met471R [17] were used to amplify the V1-V3 hypervariable regions of the 16S rRNA gene via PCR, following previously published procedures [17]. Purified archaeal amplicons (25 μL) were sent to Molecular Research DNA Laboratories (Shallowater, TX) and sequenced with the Illumina MiSeq version 3 NGS platform.

### Bioinformatics workflow used to analyze MiSeq sequences

The program MOTHUR, version 1.33.3, was used to perform bioinformatics analyses in-house [25]. Each time period was analyzed separately because of the lack of computing power and memory. Prior to using MOTHUR, a Perl script was used to trim the sequences to 350 bp at the reverse primer and each sequence was quality checked. All sequences with a Phred quality score of 25 or above were kept for further analyses. The command, trim.seqs removed barcodes and created a file that identified which sample belonged to which sequences.

To determine the number of unique sequences in the data set, the command unique.seqs was used. A Needleman-Wunsch pairwise alignment and an aligned reference file of known rumen methanogen 16S rRNA gene sequences were used with the command align.seqs to align the unique sequences. Chimeric sequences were identified with UChime [26] and removed with MOTHUR.

In order to detect any bacterial 16S rRNA gene sequences, the sequences were classified with the 16S rRNA reference Ribosomal Database Project (RDP) files provided by MOTHUR. These files contained known bacteria and methanogen sequences from kingdom to genus taxonomic levels. The files were modified to contain species-level names. Any bacteria sequences (<0.01 %) were removed. The online RDP Classifier was used at a 95 % confidence threshold to further quality check the sequences. Sequences with an unknown root were removed. Taxonomy and fasta files containing 765 known archaeal species names and 16S rRNA gene sequences were used to classify the sequence reads into taxonomic species. The command cluster.split, was used with a 2 % cutoff to cluster the 16S rNA gene sequences into OTUs. Once OTUs were formed, they were classified with the command, classify.otu. The command summary.single calculated OTU-based alpha diversities, Shannon Diversity Index, Chao I richness estimator, Inverse Simpson index, and Good’s coverage.

The subsample parameter in MOTHUR was used to analyze the same number of sequences per individual sample. Shared OTUs within and between breeds were counted with get.sharedseqs. To correlate OTUs (e.g., OTU 1 to OTU 2 at 3 DIM), the otu.association command used a Pearson correlation. The command, merge.file combined the fasta files of unique sequences. A subsample of 100,000 sequences was taken and the distances between sequences with a 2 % cutoff were calculated with dist.seqs. Once a phylip distance file was created, the clearcut command created a phylogenetic tree file. The output from clearcut was used with Fast Unifrac to perform a PCoA [27]. The PCoA determined if sequences from each sample cluster were based on breed or DIM.

### Statistical analyses

All data were analyzed with a repeated measures ANOVA model in SAS 9.4 (SAS Inst. Inc., Cary, NC) with PROC MIXED. The model included breed, DIM, and breed by DIM interactions as fixed effects and used the Kenward-Roger method to determine the degrees of freedom. A Pearson correlation, to determine the relationship between VFA and methanogen taxa, was performed with the PROC CORR. The online data visualization tool, Plotly, was used to generate a heatmap of the correlation values (r). Statistical significance was declared at *P* < 0.05 and trends were declared at 0.05 ≤ *P ≤* 0.10.

### Ethics approval and consent to participate

The present study was performed in accordance with the Institutional Animal Care and Use Committee at the University of Vermont under protocol # 13–031.

### Availability of supporting data

The data sets that support the results of the present study are included within the journal article.

## Abbreviations

DIM: days in milk
DM: dry matter
DMI: dry matter intake
H: Holstein
J: Jersey
*Mbr*: *Methanobrevibacter*
mcrA: methyl coenzyme M reductase A
*Msp*: *Methanosphaera*
NDF: neutral detergent fiber
NGS: next-generation sequencing
OTU: operational taxonomic unit
PCoA: principal coordinate analysis
RBB + C: repeated-bead beating plus column
RDP: Ribosomal Database Project
RO: *ruminantium-olleyae*
SGMT: *smithii*-*gottschalkii*-*millerae*-*thaueri*
TMR: total mixed ration
UVM: University of Vermont
VFA: volatile fatty acids
X: Holstein x Jersey crossbreed
